# Research on Fuel Offset Control of High-Pressure Common-Rail Diesel Engine Based on Crankshaft Segment Signals

**DOI:** 10.3390/s22093355

**Published:** 2022-04-27

**Authors:** Yuhua Wang, Guiyong Wang, Guozhong Yao, Lizhong Shen

**Affiliations:** Yunnan Province Key Laboratory of Internal Combustion Engines, Kunming University of Science and Technology, Kunming 650500, China; wangyuhua@stu.kust.edu.cn (Y.W.); yaoguozhong@kust.edu.cn (G.Y.); shenlizhong@kust.edu.cn (L.S.)

**Keywords:** crankshaft fragment signal, fuel supply control, fuel offset, operating uniformity

## Abstract

This paper studies the fuel supply offset of diesel engines based on the crankshaft segment signal. Engine nonuniformity refers to the crankshaft torque fluctuation caused by cylinder-to-cylinder differences caused by misfiring or differences in the fuel supply or air supply. Fuel injection offset can reduce the nonuniformity between cylinders to realize high-efficiency and low pollution combustion. Based on crankshaft segment signal characteristics, an individual cylinder fuel offset control (FOC) strategy was built. The high-pressure common-rail diesel engine operating nonuniform control strategy was put forward. Based on crankshaft segment signal characteristics at different operating conditions, the reasonable operating condition of detecting the engine individual cylinder nonuniform degree was put forward. The open-loop and closed-loop control mode based on the condition was set up. The proportional-integral (PI) control algorithm is proposed to quantify engine individual cylinder nonuniform degree, and the fuel amount offset value was obtained. According to the principle of FOC, based on the automotive electronics development ASCET software platform, the FOC strategy module of the electronic control unit (ECU) was designed, and the simulation experiment was carried out. Research shows that for Z cylinder engine, just the first Z/2 harmonic components below fire frequency can fully reflect the state of the engine’s nonuniform operation. The control target to individual cylinder FOC is zero for the synthetic waveform amplitude of the first Z/2 harmonic components. Compared with the traditional quantization method, the fuel offset information extracted from the crankshaft segment signal has stronger anti-interference and more accurate parameters. FOC algorithm can accurately reflect the engine’s operating nonuniformity. The control of the nonuniformity is reasonable. The offset fuel amount calculated by FOC is very consistent with the fuel supply state of each cylinder set by the experiment, which meets the requirement of accurate fuel injection control of the diesel engine.

## 1. Introduction

Because of increasingly serious energy consumption and environmental pollution problems, diesel engines need to use accurate electronic control technology to meet increasingly strict emission regulations and fuel consumption standards [1,2,3,4,5,6,7]. In recent years, high-pressure common-rail diesel injection systems have been improving, and the problem of fuel quantity calibration of fuel injection systems has become more prominent [8,9,10,11,12,13]. It is important to control the fuel quantity accurately to improve the performance of diesel engines [14,15,16,17]. Two main factors affect the engine operating uniformity of cylinder-to-cylinder engines. First, the amount of fuel supplied to each injector leads to nonuniform combustion between the cylinders. Due to the processing difference and wear of parts, there is a significant difference between the actual fuel injected into the cylinder and the planned fuel injection. The nonuniform fuel supply deteriorates the fuel injection control so that the diesel engine cannot produce the same torque in all cylinders. Even if the injection time is the same, the torque contribution of every cylinder is different [18,19]. Second, several other factors lead to the differences in the combustion process of each cylinder, such as differences in the processing, manufacturing of each cylinder, differences in the quality of diesel fuel, nonuniform pipe intake, etc.

The nonuniformity of the engine is a common problem. The misfire fault of the engine is an extreme condition of nonuniformity of the internal combustion engine [20,21,22,23,24,25,26,27]. However, the real-time control of internal combustion engine operation nonuniformity has not been paid as much attention as misfire detection [28,29,30,31,32]. There is little research on the control of engine nonuniformity operation. At present, the commonly used research method is to measure the operating uniformity of diesel engine by crankshaft angular speed. The crankshaft angular speed measurement of the engine is very convenient and economical [33,34,35,36,37,38,39]. Crankshaft angular speed is closely related to cylinder pressure and drive torque acting on the crankshaft. At present, there are four methods to diagnose the operating uniformity of an internal combustion engine by analyzing crankshaft angular speed fluctuation signal: Reconstruction method of split cylinder torque [40,41], Waveform analysis method [42], Multi-feature synthesis method [43], Linear observer method based on DFT [44,45,46,47]. The crankshaft angular speed is usually used to measure the operating uniformity of the engine [48,49,50]. However, internal combustion engines have many structural parameters, complex models, and large computational volumes. ECU deals with the crankshaft angular speed, such as the strong need for real-time computing and great complexity, so these methods can lead to a certain degree of calculation errors and poor real-time performance [51]. This method is mainly used for misfire diagnosis with low real-time performance but cannot be used for real-time control of engine operating nonuniformity. The current research only detects the operating uniformity of diesel engines and does not calibrate it according to the operating nonuniformity of engine [52,53,54]. New research methods are needed to control the operating uniformity of diesel engines. In order to ensure the high-efficiency performance of high-pressure common-rail diesel engines, it is necessary to accurately detect and control the degree of nonuniformity in the diesel engine. A common solution is fuel offset by using a fuel calibration curve specific to each injector [55]. This solution can only solve the nonuniformity of operation due to differences in injector manufacturing, but it cannot solve the engine’s nonuniform operation due to other factors. Therefore, it must be considered to establish an automatic fuel balance method that can diagnose and control the working unevenness of high-pressure common-rail diesel engines online.

This paper proposes an FOC control strategy based on crankshaft segment signals for the fuel compensation control of the engine operating uniformity. The crankshaft segment signal is the period of time value corresponding to several continuous crankshaft angle increment signals. The measurement principle and process of engine crankshaft segment signal [56] and instantaneous speed are the same. Crankshaft segment signal is a direct measurement parameter, and its signal acquisition is more convenient, efficient, and stable. Crankshaft segment signal can provide an accurate quantitative standard for FOC. Firstly, the characteristic operating nonuniform quantification method based on crankshaft segment signals and the FOC algorithm was proposed. Secondly, the operating uniformity control strategy of the high-pressure common-rail diesel engine under different working conditions is proposed. Finally, the proportional-integral (PI) control algorithm is proposed to quantify engine individual cylinder nonuniform degree, and the fuel amount offset value was obtained. Based on the automotive electronics development ASCET software platform, the FOC strategy module of the electronic control unit (ECU) was designed, and the simulation experiment was carried out. The experimental results show that the simulation results show that the offset fuel quantity calculated by FOC is consistent with the fuel supply state of each cylinder set by the experiment, FOC algorithm based on the crankshaft segment signal is an effective fuel offset control method for engine operating nonuniformity. This study makes the following contributions:

1. The characteristics of the crankshaft segment signal were analyzed. It is found that the degree of nonuniformity in engine operation can be represented by the amplitudes of the lower-order nondominant harmonics of the crankshaft segment signal below the firing frequency. It is a new method to measure the operating nonuniformity. It replaces the method of crankshaft angular speed. The crankshaft segment signal can be used as the basis of fuel quantity offset.
2. A method based on band-pass filtering synthetic waveform to extract the signal of nonuniform engine operation was proposed. The crankshaft segment signal extracted by this method can reduce the interference caused by the external torque factor of the engine, and the obtained parameters can more accurately reflect the nonuniformity of the engine. Compared with the crankshaft angular speeds, the result obtained by this method is more accurate. ECU is simpler and faster in calculating crankshaft segment signals.
3. In order to make the diesel engine operate uniformly, fuel offset is carried out for the nonuniform phenomenon, fuel offset is carried out for the nonuniformity phenomenon, the FOC control strategy based on the characteristics of crankshaft segment signals is proposed, and the fuel compensation value is obtained. The proposed fuel offset control method based on crankshaft segment signals can realize the online control and automatic calibration of diesel engine operating uniformity.

## 2. Crankshaft Segment Signals Acquisition and Analysis

### 2.1. Crankshaft Segment Signal Acquisition and Processing

**Figure 1:**
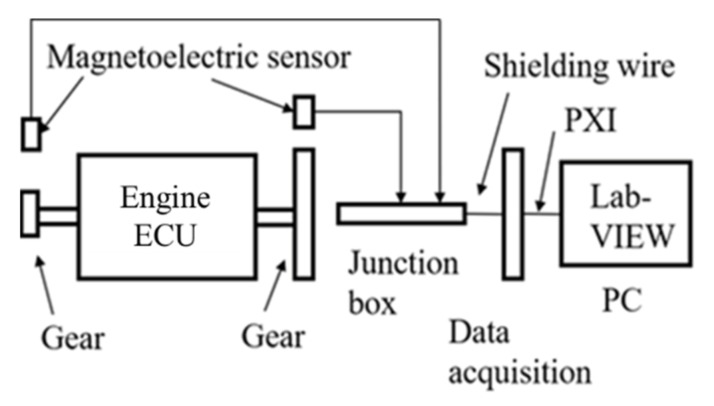
The experimental acquisition system.

The crankshaft and camshaft signals were collected synchronously in the experiment. The purpose of collecting the camshaft signal was to determine the phase of the crankshaft signal accurately. The layout of the experimental acquisition system is shown in Figure 1.

**Figure 2:**
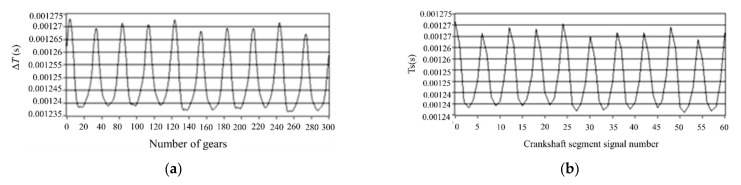
Crankshaft fragment signals containing different crankshaft delta signals. (**a**) An incremental crankshaft signal is taken as the crankshaft segment signal length Δ*T*. (**b**) Five incremental crankshaft signals are the signal length of crankshaft segment T_S_.

**Figure 3:**
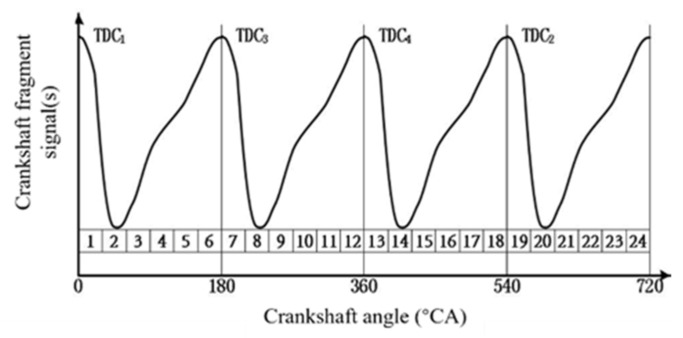
An operating cycle is divided into 24 angular segments.

A four-cylinder four-stroke high-pressure common-rail diesel engine of the model “YN30CR” (Kunming, China) was used in the experiment. The circuit design was carried out in the external ECU circuit of a single-chip microcomputer, and the module signal of the magnetoelectric sensor is converted into a square wave signal first. Then the digital Input/Output (I/O) is used to collect the crankshaft incremental signal and crankshaft segment signal. The length of the crankshaft segment signal was determined as five incremental crankshaft signals. The waveform of crankshaft segment signal with one and five-times crankshaft increment signal, respectively, is shown in Figure 2. The sampling section is lowered according to the above method in the fuel offset control of a four-cylinder engine. Each cycle consists of 24 crankshaft angular segments, shown in Figure 3.

The DFT and its inverse transform can be used to extract the lowest second-order synthetic waveform below the firing frequency in the study of crankshaft segment signals. In the software module of ECU, such computation is quite large, so it is not possible to use the same method to extract the lowest order synthetic waveform. The solution is digital bandpass filtering for standard deviation signal d_Me_. The 0.5-order and 1-order waveforms of the standard deviation signal d_Me_ are extracted respectively and then synthesized in phase to obtain the lowest second-order synthetic waveform. The digital filter used is the Infinite Impulse Response (IIR) digital Butterworth filter for band-pass filtering. The Butterworth filter is characterized by a maximum flat amplitude characteristic in the passband and the stopband, which meets the needs of the FOC band-pass filter.

Using LabVIEW’s Digital Filter Design (Filter Design) to design the IIR parameters, the transfer function of the IIR Butterworth digital filter of the crankshaft segment signals of the second order is obtained.

(1) $$H(z)=\frac{0.00034421-0.0006884Z^{-2}+0.00034421Z^{-4}}{1-3.92555566Z^{-1}+5.80000050Z^{-2}-3.82254895Z^{-3}+0.94821739Z^{-4}}$$

Into a difference equation

(2) $$\begin{array}{l} y_{4}=0.00034421x_{4}-0.00068842x_{2}+0.00034421x_{0}+3.92555566y_{3}\\ -5.80000050y_{2}+3.82254895y_{1}-0.94821739y_{0} \end{array}$$

The transfer function of the first-order crankshaft segment signal is obtained

(3) $$H(z)=\frac{0.00039699-0.00079398Z^{-2}+0.00039699Z^{-4}}{1-3.85702600Z^{-1}+5.66275721Z^{-2}-3.74833351Z^{-3}+0.94444439Z^{-4}}$$

Into a difference equation

(4) $$\begin{array}{l} y_{4}=0.00039699x_{4}-0.00079398x_{2}+0.00039699x_{0}\\ +3.85702600y_{3}-5.66275721y_{2}+3.74833351y_{1}-0.94444439y_{0} \end{array}$$

**Figure 4:**
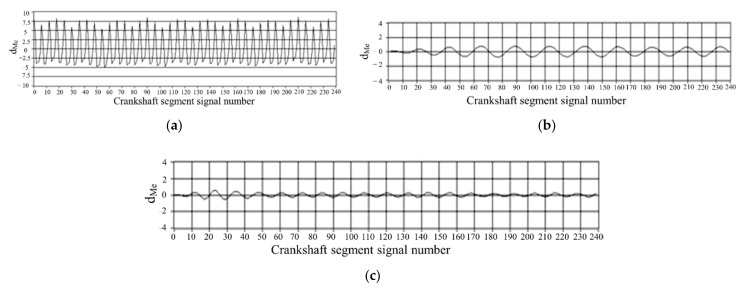
Results of two digital bandpass filters. (**a**) Bandpass filtered input signal d_Me_. (**b**) Bandpass filtering results of 0.5-order waveform. (**c**) Bandpass filtering results of 1-order waveform.

The effect of band-pass filtering is shown in Figure 4.

### 2.2. Characteristic Analysis of Crankshaft Segment Signals

**Figure 5:**
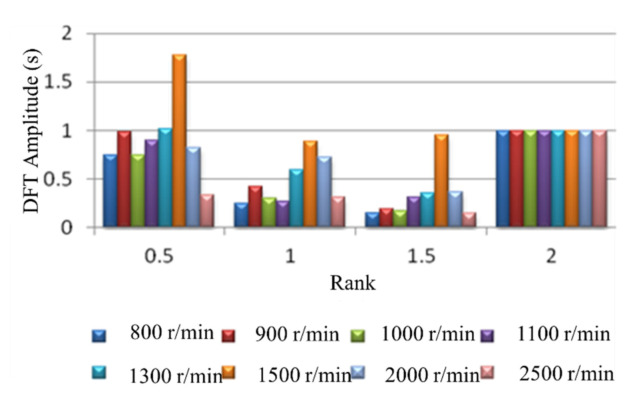
Normalized spectral amplitude at each rotational speed.

**Figure 6:**
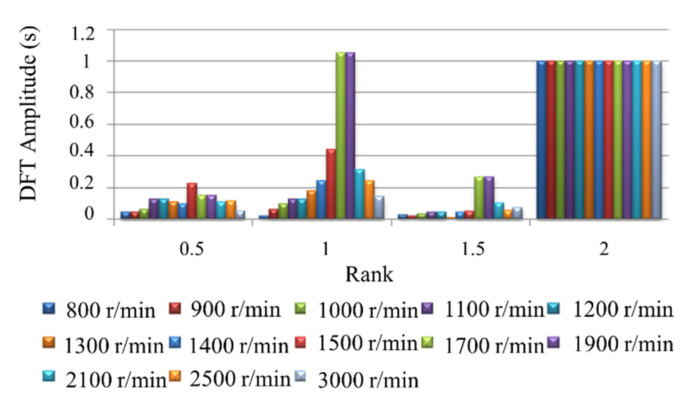
Normalized spectrum amplitude of each speed.

At the condition of engine operating nonuniformity, the signal spectrum of the crankshaft segment shows that the characteristics of the amplitude of the low-order nondominant harmonic (0.5, 1 and 1.5) significantly increased. This is an entirely different feature from that in the uniform operating condition. In order to further illustrate the above characteristics, a four-cylinder diesel engine was used in the experiment to transform the signal from the time domain to the frequency domain through the DFT. In the experiment, 1, 2, and 3 cylinders were used for normal fuel supply, and only the fourth cylinder was cut off. The 0.5, 1, 1.5 and 2 orders were selected, and the amplitude of the second-order spectrum at each rotational speed was normalized. The crankshaft segment signals’ signal waveforms and frequency spectra at different engine speeds were tested. Figure 5 shows the spectrum comparison of the crankshaft segment signals at engine operating nonuniform. Figure 6 shows the spectrum comparison of the crankshaft segment signals at engine operating uniform.

Below 2000 r/min engine speed, each rotational speed’s half order spectral amplitude is almost equal to the second-order spectral amplitude of this rotational speed. The half-order spectral amplitude of 1500 r/min rotational speed is higher than its second-order spectral amplitude. At high engine speed, the spectral amplitude of 0.5, 1 and 1.5 order harmonics is no longer obvious. This characteristic is not consistent with low and medium speeds. Because at the condition of high speed and no load, the impact of reciprocating inertia moment of every cylinder is significant, the nonuniformity operation caused by single cylinder fuel break is no longer reflected in the fluctuation of speed.

Comparing Figure 5 with Figure 6, the amplitude variation of the low-order amplitude spectrum has the most significant difference between engine running nonuniformity and uniformity. Therefore, the uniformity of engine operation is judged by the degree of change in the low-order amplitude spectrum of the engine crankshaft segment signal at low engine speed. The smaller the relative ratio of the low-order amplitude to the second-order amplitude, the more uniform the engine operating condition; the more significant the relative ratio of low-order amplitude to second-order amplitude, the more nonuniformity the engine operates.

When the engine speed exceeds 1300 r/min, the amplitude of the first-order harmonic component is higher than that of the second-order harmonic component, even at uniform operating conditions. In particular, the first-order amplitude of 1700 r/min and 1900 r/min has exceeded the second-order amplitude, which is not significantly different from the condition of nonuniformity work. Therefore, the quantitative detection of nonuniformity work cannot be carried out when the average speed exceeds 1300 r/min. Because the distortion will occur over 1300 r/min speed, uniform operating condition is misdiagnosed as the nonuniform operating condition. The magnitudes of the low-order nondominant harmonic components below the firing frequency are related to engine nonuniformity. For a Z-cylinder engine, the lowest order Z/2 harmonic components lower than the firing frequency can reflect the nonuniformity state of the engine.

## 3. Quantification of Engine Operating Nonuniformity and FOC Control Principle

### 3.1. Signal Waveform Analysis and Information Extraction of Nonuniform Engine Operation

**Figure 7:**
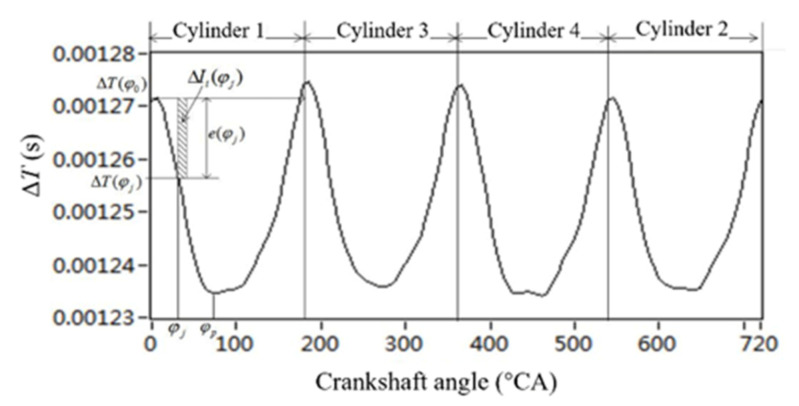
Definition of characteristic parameters of crankshaft fragment signals in Angle domain.

In steady-state engine conditions, the waveform of crankshaft segment signals in the angular domain, each operating cycle has the same waveform. Therefore, the crankshaft segment of one operating cycle is subjected to waveform analysis, as shown in Figure 7.

In the condition of uniform engine operation, the rising or falling degree of the crankshaft fragment signal in the same phase is the same. On the contrary, under the condition of nonuniform operation, the rising or falling degree is different.

To characterize the above concepts, define a parameter called position $\varphi_{j}$ of the crankshaft segment signal deviation $e\left(\varphi_{j}\right)$. $e\left(\varphi_{j}\right)$ is the difference between the crankshaft segment signal value at position $\varphi_{j}$ and the compression top dead center $\varphi_{0}$ of *i* cylinder.

(5) $$e(\varphi_{j})=[\Delta T(\varphi_{j})-\Delta T(\varphi_{0})]$$

According to the variation trends of crankshaft segment signals, it is known that in the falling range $e\left(\varphi_{j}\right)$ of crankshaft segment signals the value is negative.

The change of the cylinder 1’s crankshaft segment signal in the angle range of the engine is defined as the crankshaft angle signal parameter, expressed as $\Delta I_{i}\left(\varphi_{j}\right)$

(6) $$\Delta I_{i}(\phi_{j})=e(\phi_{j})\Delta \phi$$

Then, the sum of the angular product parameters of the crankshaft segment signals in the whole *i*-cylinder crankshaft segment signals drops range is defined as the crankshaft segment signals drop product

(7) $$I_{di}=\int_{\phi_{0}}^{\phi_{p}}e(\phi_{j})d\phi$$

where $\varphi_{p}$ is the crankshaft angular at the peak and valley position of the crankshaft segment signal in the *i*-th cylinder section, $\varphi_{0}$ is the ignition sequence of the *i*-th cylinder and the compression top dead center of the next cylinder.

The crankshaft segment signals rising product $I_{ui}$ of the crankshaft segment signals rising interval of the *i*-th cylinder can also be defined.

(8) $$I_{ui}=\int_{\phi_{p}}^{\phi_{0}}e(\phi_{j})d\phi$$

where $\varphi_{p}$ is the crankshaft angular at the peak and valley position of the crankshaft segment signal in the *i*-th cylinder section. $\varphi_{0}$ is the ignition sequence of the *i*-th cylinder and the compression top dead center of the next cylinder. However, it should be noted that the calculation base point of angular product density of crankshaft segment signal here is

(9) $$e(\phi_{j})=\left[\Delta T(\phi_{j})-\Delta T(\phi_{p})\right]$$

The main operating interval of each cylinder’s combustion gas pressure torque is the descending product or ascending product of the crankshaft segment signal in the work interval of the *i*-th cylinder in Figure 7. The nonuniformity degree of each cylinder can be indicated by comparing the difference between rising and falling intervals. For a Z-cylinder engine, the lowest order Z/2 harmonic components lower than the firing frequency can reflect the nonuniformity state of the engine. Therefore, Z/2 harmonic components of the lowest order below the firing frequency are extracted to replace the original crankshaft segment signals. For a four-cylinder engine, the two harmonic components below the firing frequency are half-order and first-order.

**Figure 8:**
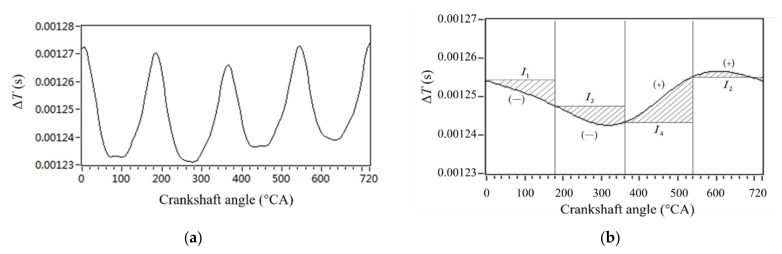
Parameter extraction of crankshaft segment signal waveform. (**a**) Crankshaft segment signals waveform. (**b**) Synthesized waveforms of 0.5 order and 1 order.

The crankshaft segment signal waveform under the nonuniform fuel supply condition of the fourth cylinder is shown in Figure 8a. The lowest two-order synthetic waveforms of order 0.5 plus 1 were obtained by DFT and IDFT, as shown in Figure 8b. The amplitude of the 0.5 order harmonic is higher than the first-order harmonic. Therefore, the lowest second-order synthetic waveform change becomes simpler for each cylinder compared to the original crankshaft segment signals.

In Figure 8b, the waveform trend of the operating interval of each cylinder reflects the comprehensive torque action result of the cylinder. Cylinder 1 is completely in the decline range, indicating that under the action of the cylinder torque, the crankshaft has been accelerating in the range of the lowest second order, which reflects the strong effect of the torque, but also reflects the relatively large fuel supply. The same is true for the fuel supply of cylinder 3. The acceleration of the crankshaft in the lowest second-order range ends in the cylinder 3 torque action interval. The crankshaft decelerates in the cylinder 4 torque action with a higher deceleration. This reflects the weaker comparative torque action of cylinder 4. Therefore, the fuel supply of cylinder 4 is less. The torque of cylinder 2 is a slight deceleration and then a slight acceleration, indicating that the fuel supply of cylinder 2 is relatively moderate.

According to Figure 8a, the difference in fuel supply offset obtained from the lowest second-order waveform analysis is completely consistent. Therefore, the 0.5-order and 1.5-order synthetic waveforms can reflect the nonuniformity between cylinders. When the amplitude of the synthetic waveform of 0.5-order and 1-order components is 0, it is the control target of FOC.

Compared with the traditional nonuniformity parameter quantization method based on the peak value difference of crankshaft angular speed fluctuation, this method has two advantages: First, the waveform of the crankshaft segment signal is processed, and the lowest Z/2 harmonic synthesis signal is obtained below the firing frequency. This method eliminates all order harmonics that have nothing to do with the nonuniformity of the engine. Therefore, this method eliminates the external torque interference of the engine, and the result can better reflect the nonuniformity of the engine. Second, the accumulation of the variation of the lowest second-order harmonic synthesis signal of the crankshaft segment signal of each cylinder is considered. The obtained parameters can more accurately reflect the operating nonuniform of the engine.

### 3.2. FOC Control Calculation Based on PI Control Principle

The fuel offset control (FOC) is carried out by using the PID control principle. The control goal of each cylinder is that the lowest Z/2 order synthetic waveform does not rise or fall, that is, the amplitude of the synthetic waveform of 0.5-order and 1-order components is 0. It is assumed that the fuel quantity is controlled after each sampling, and the control signal deviation at the angular position $\phi_{k}$ is

(10) $$e(\phi_{k})=[\Delta T_{\frac{Z}{2}}(\phi_{k})-\Delta T_{\frac{Z}{2}}(\phi_{0})]$$

where $\phi_{0}$ is the compression top dead center of cylinder I. $\Delta T_{\frac{Z}{2}}\left(\varphi_{k}\right)$ is the amplitude of the lowest Z/2 order synthetic waveform at the angular position and $\Delta T_{\frac{Z}{2}}\left(\varphi_{0}\right)$ is the amplitude of the lowest Z/2 order synthetic waveform at the angular position. According to the PID control principle, the total fuel supply is

(11) $$u(\phi_{k})=K_{P}e(\phi_{k})+K_{I}\sum_{j=0}^{k}e(\phi_{j})+K_{D}[e(\phi_{k})-e(\phi_{k-1})]$$

where $K_{P}$ is proportional coefficient; $K_{I}$ is the integral coefficient; $K_{D}$ is the differential coefficient.

Because the feedback control is carried out only once in each operating cycle of the engine, there is no need for differential control, so Equation (12) can be abbreviated as follows:

(12) $$u\left(k\right)=K_{P}e\left(k\right)+K_{I}\overset{k}{\underset{j=0}{\sum }}e\left(j\right)$$

where *k* is the sampling serial number, *k* = 0, 1, 2, … *u*(*k*) is the control value at the *K*th sampling time; *e*(*k*) is the input deviation at the *K*th sampling time.

The control output of Equation (13) is the total control quantity. To calculate the offset fuel quantity, the difference between two adjacent controls should be calculated:

(13) $$\begin{array}{l} \Delta u(k)=u(k)-u(k-1)\\ \;\;\;=\left(K_{P}e(k)+K_{I}\sum_{j=0}^{k}e(j)\right)-\left(K_{P}e(k-1)+K_{I}\sum_{j=0}^{k-1}e(j)\right)\\ \;\;\;=K_{P}[e(k)-e(k-1)]+K_{I}e(k) \end{array}$$

If there are *m* sampling points in the action range of a cylinder division, the sum of the control deviation for the cylinder division is

(14) $$\begin{array}{l} Q_{}=\sum_{k=1}^{m-1}\Delta u(k)=\sum_{k=1}^{m-1}\left\{K_{P}[e(k)-e(k-1)]+K_{I}e(k)\right\}\\ \begin{array}{ccc} & & = \end{array}K_{P}[e(m-1)-e(0)]+K_{I}\sum_{k=1}^{m}e(k) \end{array}$$

where *Q* is the compensation control oil quantity of FOC, and it is also the quantitative parameter of uneven cylinder division of the engine.

If sampling is carried out every 6 °CA, there are 30 sampling points in the operating range of the whole cylinder, and the sum of the control deviation is

(15) $$Q_{}=K_{P}[e(29)-e(0)]+K_{I}\sum_{k=1}^{29}e(k)$$

where *Q*_FOC_ is the offset control fuel quantity of FOC, and it is also the quantitative parameter of uneven cylinder division of the engine.

Because $Q_{}$ is only the sum of the control deviation quantity, it does not represent the actual offset fuel quantity in the end. The final offset fuel quantity is the product of the integral value *Q* of the control deviation quantity and the weight value *W* of the fuel quantity.

The weight is divided into two types, fuel weight *W_q_* and speed weight *W_n_*, and the final weight *W* is obtained by multiplying these two weights:

(16) $$W=W_{q}W_{n}$$

Therefore, the final actual offset fuel value is

(17) $$Q=\left\{K_{P}[e(29)-e(0)]+K_{I}\sum_{k=1}^{29}e(k)\right\}W_{n}W_{q}$$

where $K_{P}$ is proportional coefficient, *e*(*k*) is the input deviation at the *K*th sampling time. $K_{I}$ is the integral coefficient, *W_q_* is fuel weight, *W_n_* is engine speed.

## 4. Control Strategy of Subcylinder Fuel

### Offset Based on the Operating Condition

**Figure 9:**
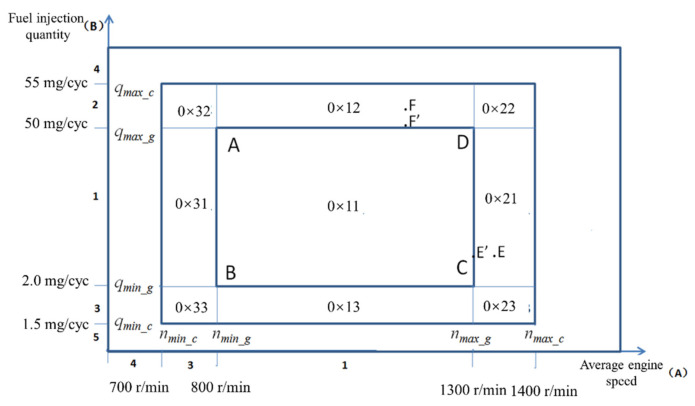
Control areas are divided according to fuel injection volume and average speed.

The nonuniform operation of the engine is most evident at low idle. FOC for cylinder uniformity should be mainly applied to the engine at low idle speed. Considering the vehicle ride comfort, the engine fuel bias control should be gradually diminished with increasing speed and load, rather than abruptly interrupted. Therefore, the control area of FOC can be divided into three parts according to the current fuel injection volume and speed of the engine: closed-loop control area, open-loop control area, and invalid area, shown in Figure 9.

In the closed-loop control area, the system detects the nonuniformity of each cylinder and performs PI integration. In addition, the offset fuel quantity for each cylinder is calculated, and the injection quantity for each cylinder is corrected with the latest calculation results. In the closed-loop control area, the detection function of cylinder uniformity can be triggered only when the engine operation is stable.

There are eight subpartitions in the open-loop control area, which can be grouped into two broad categories: the first type is that both speed and fuel quantity are in the open-loop zone, as shown in Figure 9 (0 × 32, 0 × 33, 0 × 22, and 0 × 23). The second type is that only one of the speeds and fuel quantity are in the open-loop region, and the other is in the closed-loop region.

The open-loop control area is also divided into two types of control methods. In the first type of region, when the engine speed-fuel quantity is in the 0 × 32 area, the close-loop region’s nearest point is point A, so its integral value *Q*_FOC_ is the integral value of point A. In the first type of region, when the engine speed-fuel quantity is in the 0 × 32 region, the nearest point to the closed-loop area is point A, so its integral value is the integral value of point A. Similarly, the integral values of points B, C, and D will be used for the integral values of 0 × 33, 0 × 23, and 0 × 22 open-loop regions. In the second type of region, when the engine speed-fuel is at point F, the point within the closest closed-loop zone to it is point F’. Thus the integral value of point F will be applied to the integral value of point F’, and similarly, the integral value of point E will be applied to the integral value of point E’.

In the closed-loop control area, FOC should calculate the integral value *Q*_FOC_, and the calculation process should be as short as possible to meet the real-time c. In the closed-loop control area, there is no need to calculate the weight *W*, that is, the weight corresponding to speed and fuel quantity is set to 1. FOC only needs to calculate the weight in the open-loop control region, not the integral value. Its integral value only needs to call the integral value in the nearest closed-loop region. This call process is very fast.

Fuel weight *W_q_* and speed weight *W_n_* are calculated according to the characteristics of linear change, and the specific calculation formula is

(18) $$W_{q}=\left\{\begin{array}{ll} \frac{q-q_{\min\_c}}{q_{\min\_c}-q_{\min\_c}} & q_{\min\_c}\leq q<q_{\min\_g}\\ 1 & q_{\min\_g}\leq q\leq q_{\max\_g}\\ \frac{q-q_{\max\_c}}{q_{\max\_g}-q_{\max\_c}} & q_{max\_g}<q\leq q_{\max\_c} \end{array}\right.$$

(19) $$W_{n}=\left\{\begin{array}{ll} \frac{n-n_{\min\_c}}{n_{\min\_g}-n_{\min\_c}} & n_{\min\_c}\leq n<n_{\min\_g}\\ 1 & n_{\min\_g}\leq n\leq n_{\max\_g}\\ \frac{n-n_{\max\_c}}{n_{\max\_g}-n_{\max\_c}} & n_{\max\_g}<n\leq n_{\max\_c} \end{array}\right.$$

where *q* is the current fuel quantity; *n* is the current average speed; $q_{\min\_c}$ is the minimum fuel quantity in the control state; $q_{\min\_g}$ is the minimum fuel quantity in the management state; $q_{\max\_c}$ is the maximum fuel yield in the control state; $q_{\max\_g}$ is the maximum oil yield in the management state; $n_{\min\_c}$ is a set point for FOC control of low idle speed; $n_{\min\_g}$ for FOC management low idle speed set point speed; $n_{\max\_g}$ is the maximum speed of the management state; $n_{\max\_c}$ is the maximum speed of the control state.

In the invalid operating area, the system stops all functions of balancing the uniformity of the cylinders, that is, no testing or modification shall be carried out. The FOC will be frozen or its function will be closed if the engine fuel and speed are not in the range of speed-fuel in the open and closed loops. The system will not carry out signal processing for crankshaft segments that consumes computing resources.

## 5. Control Strategy Simulation Experiment Verification

According to the quantified parameters of the operating uniformity of each cylinder, the balance control operating uniformity of each cylinder is carried out. The basic idea of the control is to control FOC by fuel offset aiming at the torque balance of each cylinder. In the design, LabVIEW software was used for system design and parameter setting, and electronic control software was developed based on ASCET.

**Figure 10:**
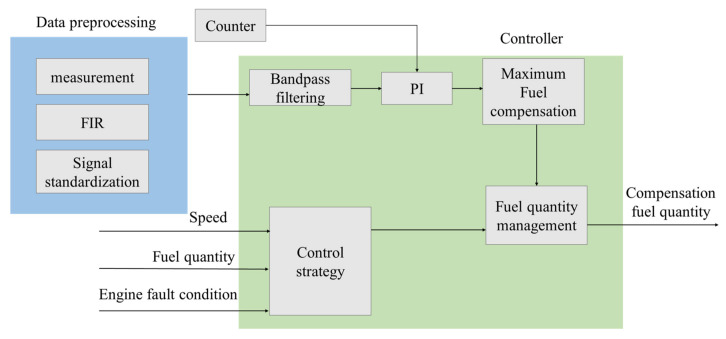
Structure of operating uniformity control strategy scheme.

The FOC control strategy scheme is shown in Figure 10. The FOC control strategy is divided into two parts: data preprocessing and controller.

**Figure 11:**
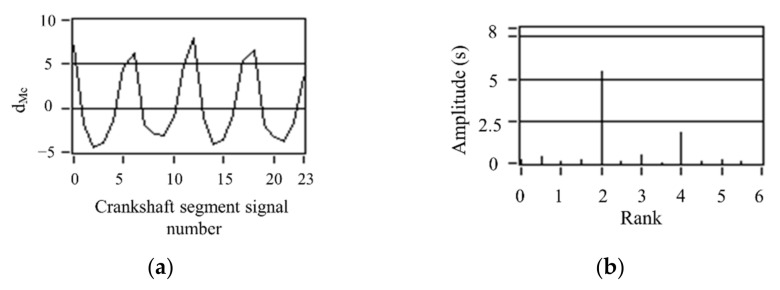

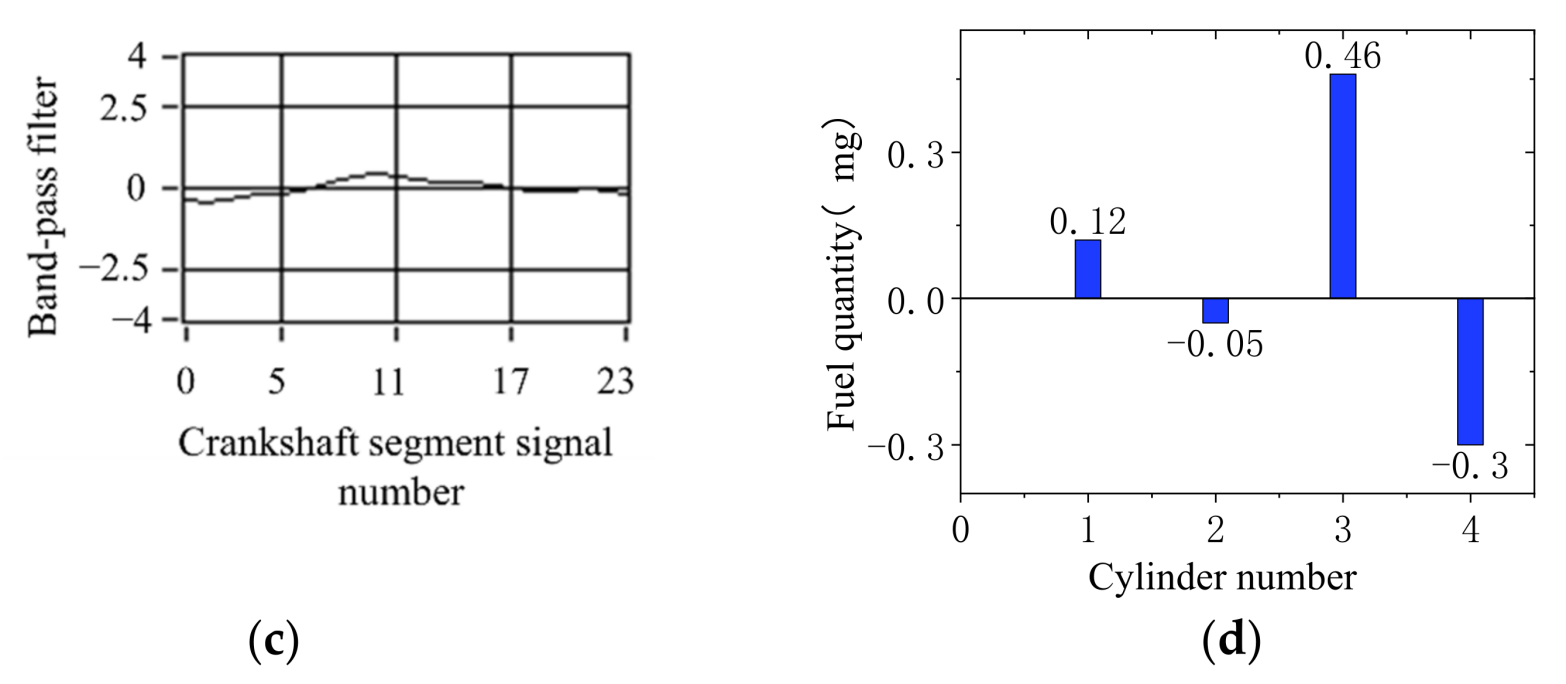
Simulation results of uniform operation (800 r/min, 0 N·m). (**a**) Deviation normalized signal dMe. (**b**) spectrum. (**c**) The two bandpass filters are added. (**d**) Compensation fuel quantity. (**c**) The two bandpass filters are added. (**d**) Offset fuel quantity.

**Figure 12:**
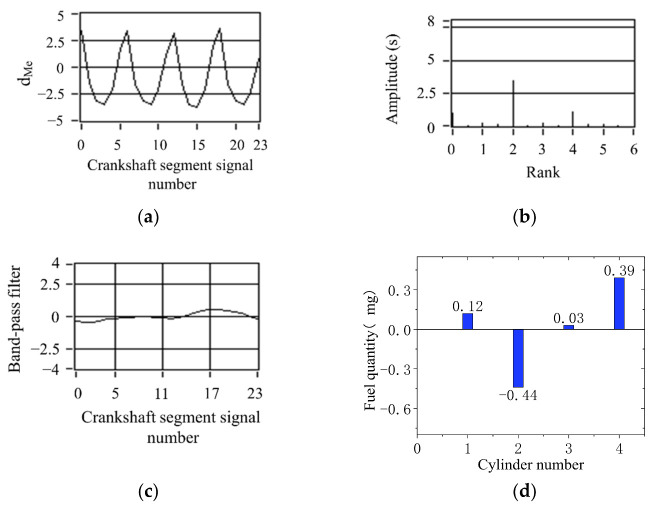
Simulation results of uniform operation (1000 r/min, 40 N·m). (**a**) Deviation normalized signal dMe; (**b**) spectrum; (**c**) the two bandpass filters are added; (**d**) offset fuel quantity.

According to the quantization method of operating nonuniformity and FOC control strategy, the FOC controller module was developed based on ASCET software. ASCET is a tool for the model-based development of embedded system application software. The fluctuation trends of crankshaft segment signals and FOC calculation results in the condition of uniform engine operation are studied. First, the crankshaft segment signal was measured in the experiment. Secondly, DFT was used to transfer the signal from the angular domain to the frequency domain, revealing the fluctuation patterns of the crankshaft signal at different operating conditions in detail, obtaining each frequency component’s proportion, and identifying the main fluctuation component. Finally, the 0.5-order and 1.5-order bandpass filtered waveforms are superimposed to obtain the synthetic waveform, as shown in Figure 11c and Figure 12c. At the condition of uniform operation, the waveform is stable, and the offset relative to *y* = 0 is slight. The waveform signals in the angular domain and frequency domain waveforms are conditions shown in Figure 11a,b and Figure 12a,b. In the uniform operation, the results of the FOC control strategy simulation calculation are shown in Figure 11 and Figure 12d. The absolute value of each cylinder’s calculated offset fuel does not exceed 0.5. Therefore, the proportionality factor *K*_p_ and the integration factor *K*_I_ are chosen correctly. In the experiment, when the maximum value of calculated FOC for each cylinder is less than 0.5 mg, all cylinders stop fuel offset. The results show that the simulation is consistent with the experimental setting.

**Figure 13:**
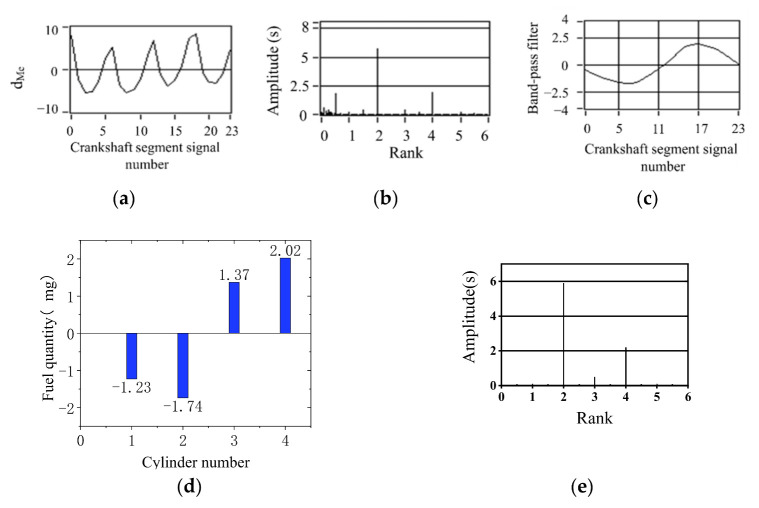
Simulation results of 80% fuel supply of the cylinder 4 (800 r/min, 0 N·m). (**a**) Deviation normalized signal d_Me_; (**b**) spectrum; (**c**) the two bandpass filters are added; (**d**) offset fuel quantity. (**e**) spectrum after fuel compensation.

**Figure 14:**
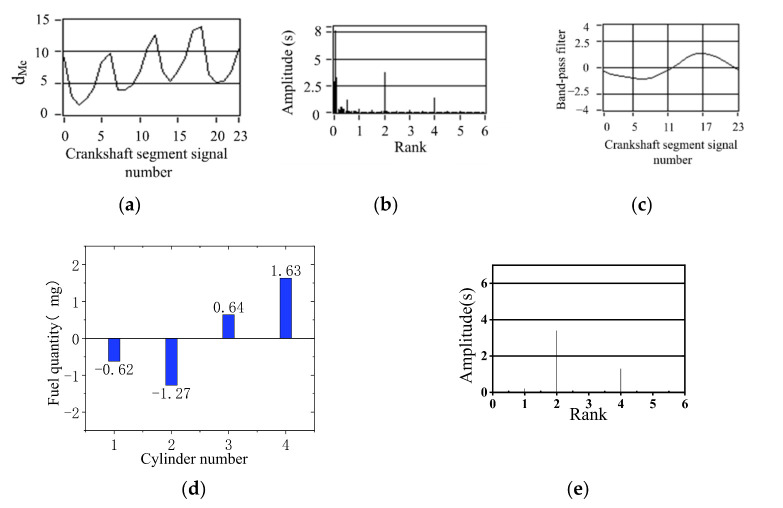
Simulation results of 80% oil supply of the cylinder 4 (900 r/min, 0 N·m). (**a**) Deviation normalized signal d_Me_; (**b**) spectrum; (**c**) the two bandpass filters are added; (**d**) offset fuel quantity. (**e**) spectrum after fuel compensation.

**Figure 15:**
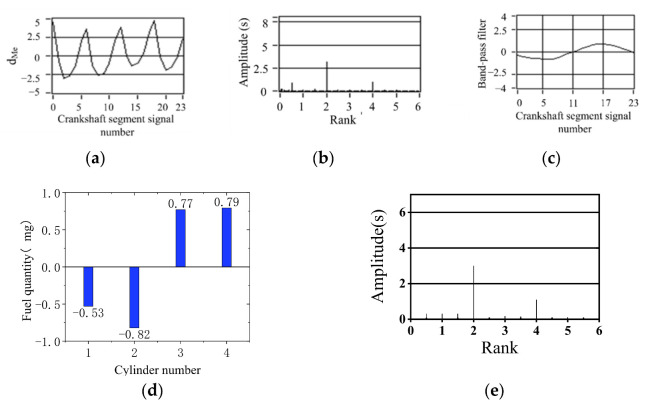
Simulation results of 80% oil supply of the cylinder 4 (1000 r/min, 0 N·m). (**a**) Deviation normalized signal d_Me_; (**b**) spectrum; (**c**) the two bandpass filters are added; (**d**) offset fuel quantity. (**e**) spectrum after fuel compensation.

Figure 13, Figure 14 and Figure 15 show the calculation results of the nonuniform operation of the engine at different speeds. These three operating conditions are in the bench test; the fourth cylinder has limited fuel supply; fuel supply is 80% of the other three cylinders; this eventually caused nonuniformity. In spectrum diagrams, the degree of nonuniformity in engine operation can be represented by the amplitudes of the lower-order nondominant harmonics of the crankshaft segment signal below the firing frequency. It can be seen from the spectrum that there is a slight nonuniformity operation. In the synthetic waveform of the band-pass filter, the synthetic waveform is biased relative to *y* = 0. The simulation results show that the fuel offset calculation result of the fourth cylinder is the largest, which accords with the actual situation of the experiment. The increase and decrease in the remaining three cylinders show that the total offset fuel of the four cylinders is 0, so it will not cause the overall increase in the average speed. The signal of the crankshaft segment after the offset of fuel volume was measured experimentally, and the signal was analyzed by spectrum analysis. The amplitudes of 2 order and 4 order are high, and the amplitudes of 0.5 order and 1.5 order are almost 0, as shown in Figure 13e, Figure 14e and Figure 15e. The signal fluctuation trend is consistent with the crankshaft segment signals spectrum variation characteristics in the uniform engine operation. Therefore, the fuel offset results calculated by the simulation are accurate.

**Figure 16:**
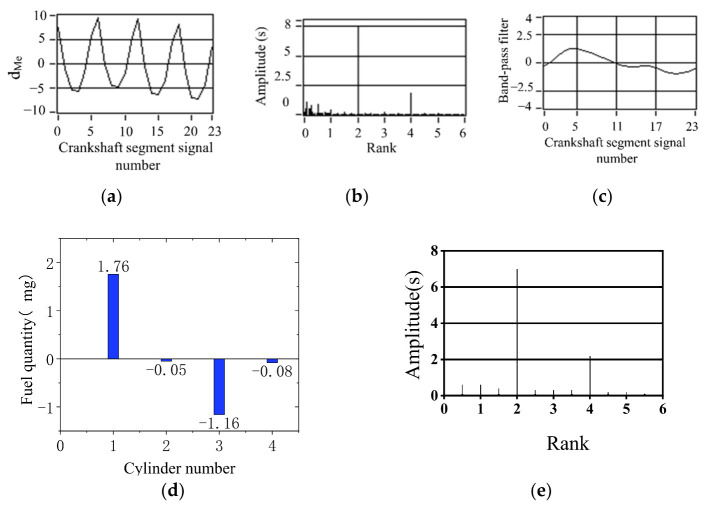
Simulation results of 90% fuel supply of the first cylinder (800 r/min, 40 N·m). (**a**) Deviation normalized signal d_Me_; (**b**) spectrum; (**c**) the two bandpass filters are added; (**d**) offset fuel quantity. (**e**) Spectrum after fuel compensation.

**Figure 17:**
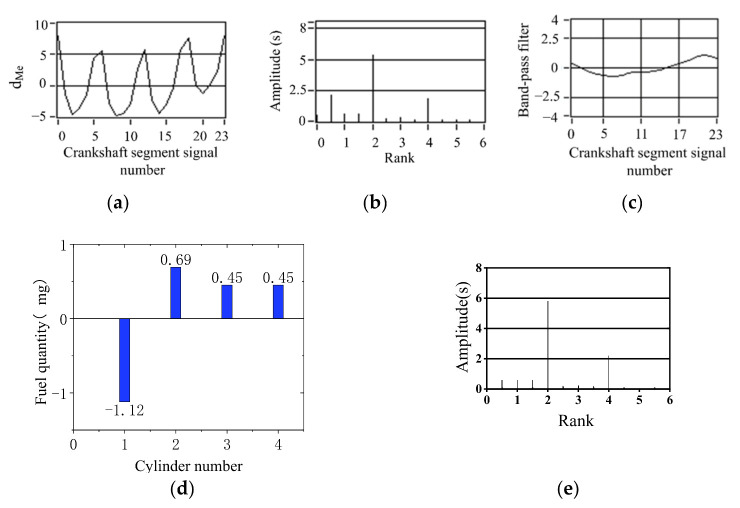
Simulation results of 90% fuel supply of the second-cylinder (800 r/min, 0 N·m). (**a**) Deviation normalized signal d_Me_; (**b**) spectrum; (**c**) the two bandpass filters are added; (**d**) offset fuel quantity. (**e**) Spectrum after fuel compensation.

**Figure 18:**
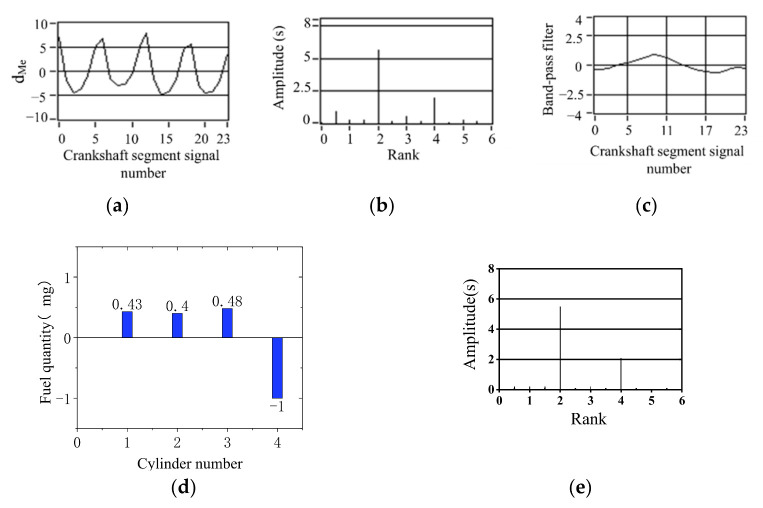
Simulation results of 90% fuel supply for cylinder 3 (800 r/min, 0 N·m) (**a**) Deviation normalized signal dMe; (**b**) spectrum; (**c**) the two bandpass filters are added; (**d**) offset fuel quantity. (**e**) Spectrum after fuel compensation.

The simulation results of 90% fuel supply of the cylinder 1 are shown in Figure 16. The simulation results of 90% fuel supply of the cylinder 2 are shown in Figure 17. The simulation results of 90% fuel supply for cylinder 3 are shown in Figure 18. The maximum value of offset fuel can always correspond to the specific cylinder set in the experiment test. This remains true after changing the engine load. The larger load of 40 N m is at 800 r/min speed, and the remaining two operating conditions of no load are shown in Figure 16. The signal fluctuation trend is consistent with the crankshaft segment signals spectrum variation characteristics in the uniform engine operation after fuel offset. The experiments verify that the simulated fuel offset results are accurate.

**Figure 19:**
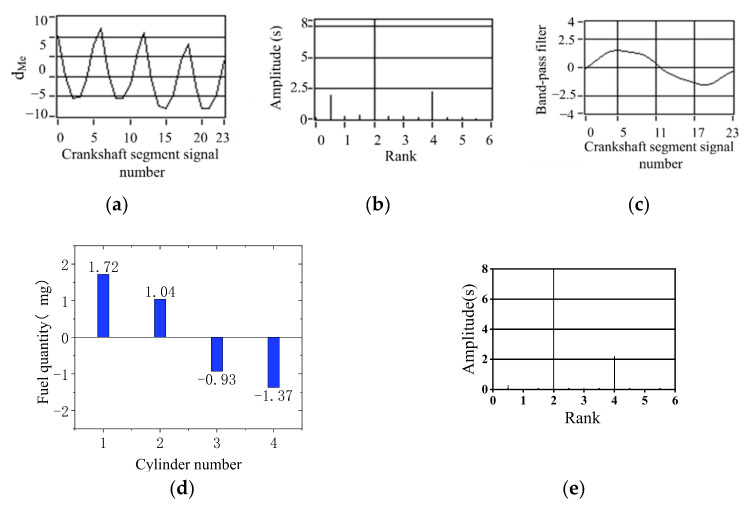
Cylinder 1 and cylinder 2 supply 90% oil (800 r/min, 60 N·m) (**a**) Deviation normalized signal d_Me_; (**b**) spectrum; (**c**) the two bandpass filters are added; (**d**) offset fuel quantity. (**e**) Spectrum after fuel compensation.

**Figure 20:**
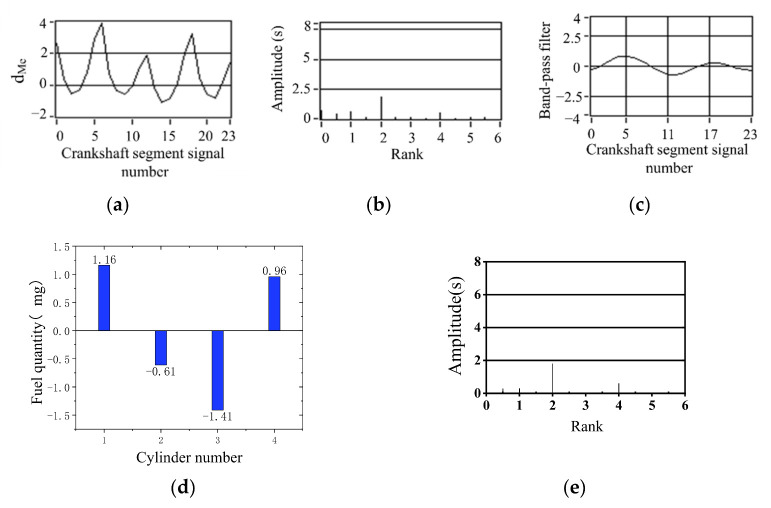
The cylinder 1 and cylinder 4 supply 90% oil (1300 r/min, 100 N·m) (**a**) Deviation normalized signal d_Me_; (**b**) spectrum; (**c**) the two bandpass filters are added; (**d**) offset fuel quantity. (**e**) Spectrum after fuel compensation.

The condition of limiting fuel supply for cylinder 1 and cylinder 2 simultaneously, with a load of 60 N·m, is shown in Figure 19. The condition of limited fuel supply for cylinder 1 and cylinder 4 simultaneously, with a load of 100 N·m, is shown in Figure 20. The figure shows the corresponding band-pass filtering and the corrected fuel after integral proportion calculation. The signal fluctuation trend is consistent with the crankshaft segment signals spectrum variation characteristics in the uniform engine operation. Therefore, the fuel offset results calculated by the simulation are accurate.

After experimental verification, the fuel offset results calculated by simulation are accurate. FOC strategy has significant advantages in accuracy and stability. There are two reasons: First, it can be seen from the spectrum that there is a large amplitude at very low order, which is caused by the fluctuation of average speed. Second, when the engine speed increases, a new trough will appear on the crest of the instantaneous rotating speed with the rise in the reciprocating inertia force. Because of these two reasons, it is not reliable to calculate the nonuniformity directly by the waveform peak value or valley value of instantaneous speed. The band-pass filtering method of FOC is not disturbed by low-frequency fluctuation and reciprocating inertia force fluctuation. It only extracts the components in 0.5 order and 1.5 order the fundamental frequency caused by the nonuniform operation of each cylinder, so it has good accuracy and reliability.

## 6. Conclusions and Future Directions

The main conclusions of this study can be summarized as follows.

(1) Based on the band-pass filtering method, the signal reflecting the nonuniformity of engine operation is extracted from the crankshaft segment signal, which is more resistant to interference than the traditional method of quantifying the nonuniformity parameter constructed based on the crest difference of instantaneous speed fluctuation, and the obtained parameter can accurately reflect the nonuniformity.
(2) For a Z-cylinder engine, the lowest order Z/2 harmonic components lower than the firing frequency can fully reflect the nonuniformity state of the engine. According to the amplitude of the lowest Z/2 order, synthetic waveform is 0. A proportional-integral (PI) algorithm is proposed to quantify the working nonuniformity of each cylinder. The quantitative parameters of the working nonuniformity of each cylinder are obtained.
(3) Based on the quantization parameters of the crankshaft segment signal, a reasonable working condition was proposed to detect the engine operating nonuniformity, and the open-loop and closed-loop cylinder fuel offset control algorithm was established.
(4) The simulation results show that the offset fuel quantity calculated by FOC is consistent with the fuel supply state of each cylinder set by experiment. FOC algorithm based on crankshaft segment signal is an effective fuel offset control method for engine operating nonuniformity.

## Data Availability

The study did not report any data.
